# Supplementary figures and images for: In utero exposure to protease inhibitor-based antiretroviral regimens delays growth and developmental milestones in mice
Source: PLoS One. 2020 Nov 19;15(11):e0242513. doi: 10.1371/journal.pone.0242513 (PMC7676697; doi:10.1371/journal.pone.0242513)

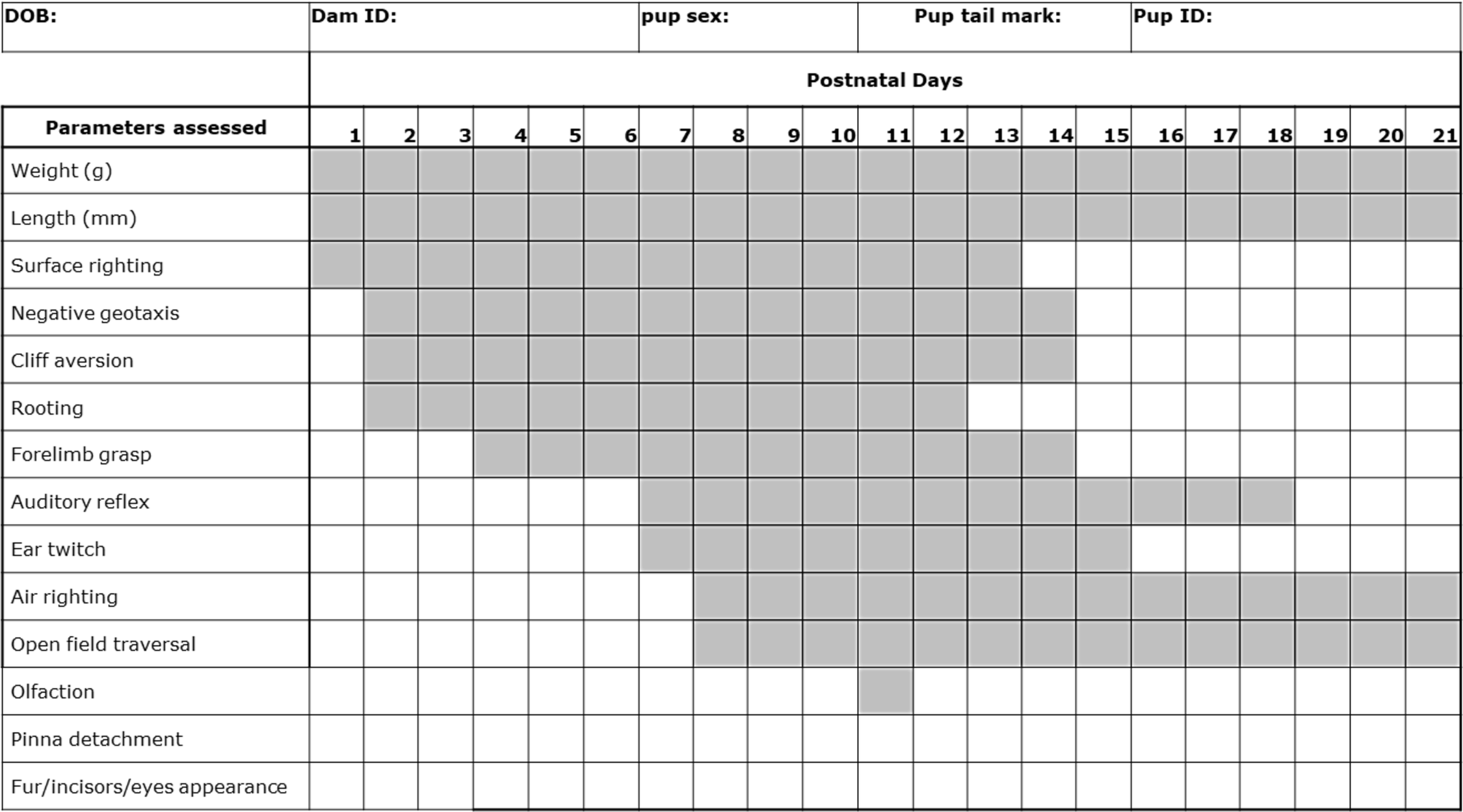

Supplement: S1 Fig — The shaded boxes mark the tests that were performed on each day. Tests were done in a sequence that was age appropriate for mice pups. (TIF) [file pone.0242513.s001.tif]
